# Emergency colonoscopy for rectal ulcer bleeding: argon plasma coagulation failure rescued by underwater snare-tip coagulation and self-assembling peptide hydrogel

**DOI:** 10.1055/a-2772-0311

**Published:** 2026-01-15

**Authors:** Ludovico Alfarone, Simone Dibitetto, Antonio Capogreco, Davide Massimi, Roberta Maselli, Cesare Hassan, Alessandro Repici

**Affiliations:** 1 9268 Endoscopy Unit, IRCCS Humanitas Research Hospital, Rozzano, Milan, Italy; 2 437807 Department of Biomedical Sciences, Humanitas University, Pieve Emanuele, Milan, Italy

Lower gastrointestinal bleeding represents a significant clinical challenge 1 2 , particularly in patients receiving antithrombotic therapy. Management requires prompt identification of the bleeding source and appropriate endoscopic intervention. The use of dual antiplatelet agents and anticoagulation increases both the risk and the severity of bleeding, complicating therapeutic decisions 3 .

**Fig. 1:**
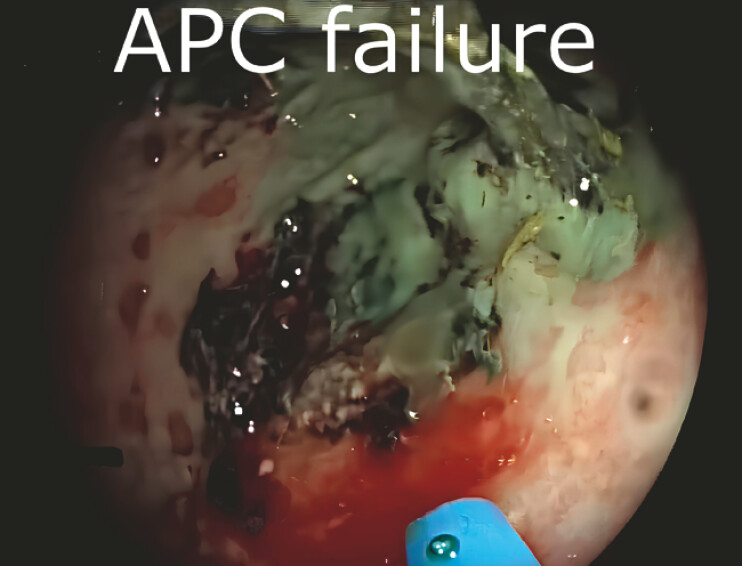
Argon plasma coagulation (APC) attempt failed to achieve hemostasis due to the deeply located bleeding vessels within the rectal ulcer base.

**Fig. 2:**
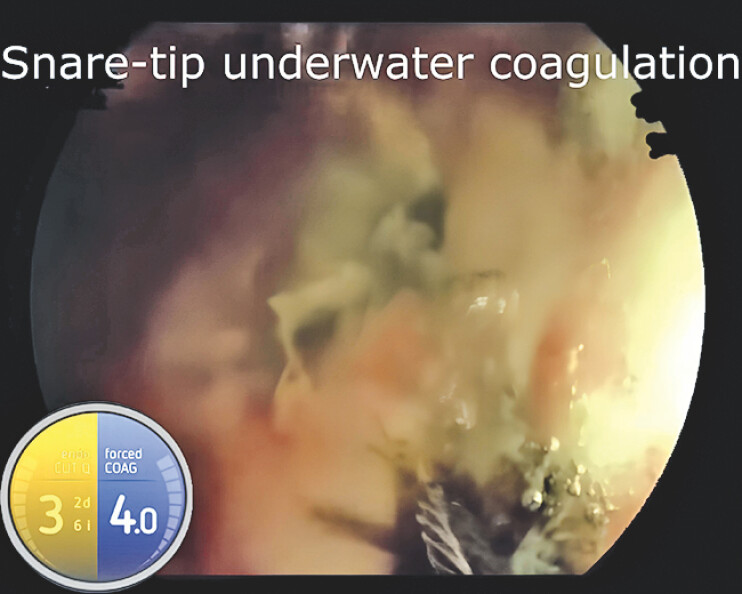
Underwater snare-tip forced coagulation (forced COAG 4.0) successfully achieved hemostasis by allowing stable vessel contact and precise energy delivery at the ulcer base.

**Fig. 3:**
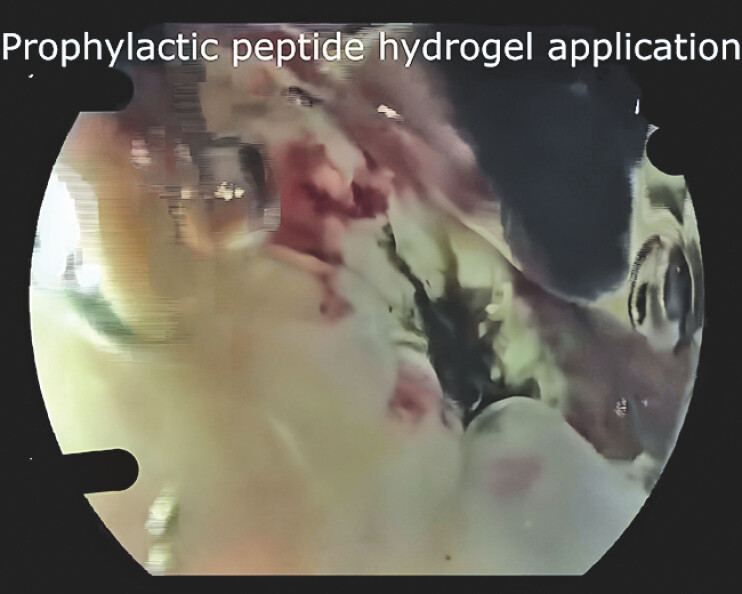
Application of the self-assembling peptide hydrogel over the treated ulcer area to promote mucosal healing and prevent rebleeding after successful hemostasis.

We describe the case of 72-year-old patient who had recently undergone radical cystectomy and nephroureterectomy for urothelial carcinoma. The postoperative course was complicated by an ST-elevation myocardial infarction (STEMI) due to the patient’s frailty, revascularization was not feasible, and therefore dual antiplatelet therapy along with low molecular weight heparin was initiated. Shortly thereafter, the patient presented with severe rectal bleeding requiring blood transfusion. Colonoscopy revealed a circumferential rectal ulcer covered by a large adherent clot. Following the removal of the clot with a snare, active oozing bleeding from a visible vessel was identified at the ulcer base. Hemostasis with argon plasma coagulation (APC) was attempted but failed because the bleeding was deeply located and the energy delivery was insufficient to achieve effective coagulation ( Fig. 1 ). Then, underwater snare-tip forced coagulation (forced COAG 4.0) was performed, allowing stable vessel contact and controlled energy delivery, successfully achieving complete hemostasis ( Fig. 2 ). A layer of self-assembling peptide hydrogel was subsequently applied ( Fig. 3 ) over the treated area as a prophylactic measure to promote mucosal healing and reduce the risk of rebleeding ( Video 1 ).

Endoscopic management of severe rectal bleeding in a patient on dual antiplatelet therapy and low molecular weight heparin. Underwater snare-tip coagulation successfully controls bleeding, followed by self-assembling peptide hydrogel application for healing.Video 1

This case highlights the complex management of lower GI bleeding in patients under combined antithrombotic therapy following recent major surgery and myocardial infarction. The underwater snare-tip coagulation technique provided an effective and safe solution when conventional APC was ineffective, offering enhanced visualization and precise thermal control. Adjunctive self-assembling peptide hydrogel application supported mucosal stabilization. This report underscores the importance of adaptable endoscopic approaches in managing high-risk bleeding in patients requiring intensive antithrombotic therapy.

Endoscopy_UCTN_Code_TTT_1AQ_2AZ
